# Optimum efficiency of treatment plants discharging wastewater into river, case study: Tigris river within the Baghdad city in Iraq

**DOI:** 10.1016/j.mex.2017.10.009

**Published:** 2017-10-31

**Authors:** Tariq J. Al-Musawi, Ibrahim A. Mohammed, Huda M. Atiea

**Affiliations:** a Isra University, Faculty of Engineering, Department of Civil Engineering, Amman, Jordan; b University of Kufa, College of Engineering, Department of Civil Engineering, Najaf, Iraq

**Keywords:** The optimization methods: of linear programming, Management, Optimization, Linear, Wastewater, Efficiency, MATLAB

## Abstract

The present study aims to manage and determine the most economical efficiency of five wastewater treatment plants discharging wastewater into the Tigris River in Iraq. The management system was based on ensuring the five-day biological oxygen demand concentration in the river is <30 mg/L according to the Iraqi standards. In many cases, the determined optimized efficiencies were found to be lower than the present working efficiencies. Although this was good for the environment, it was not cost-effective. This study revealed that the variation of river flow rates was not an important factor that effects on the results obtained. It was found that the variation of organic decomposition value in the river and the minimum efficiency limit of the first upstream plant greatly affected the operating efficiency of the downstream plants. Furthermore, no constant rank was recorded for the effects of the natural decomposition on the operating efficiency of each plant. Three points were highlighted from this study:

• The optimization methods were used to determine the most economical efficiency of multi wastewater treatment plants.
• The effects of the BOD decomposition value, the river flow, and the minimum efficiency limit were also investigated.
• This study presents the linear modeling method in detail and has a scientific impact for similar studies.

## Graphical abstract

**Figure:**
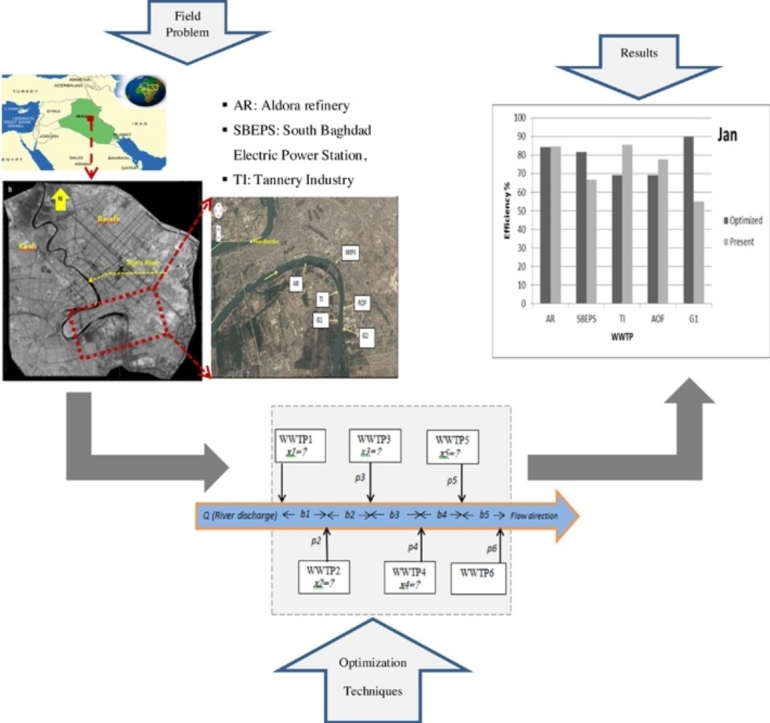

## Method details

### Study area

**Fig. 1:**
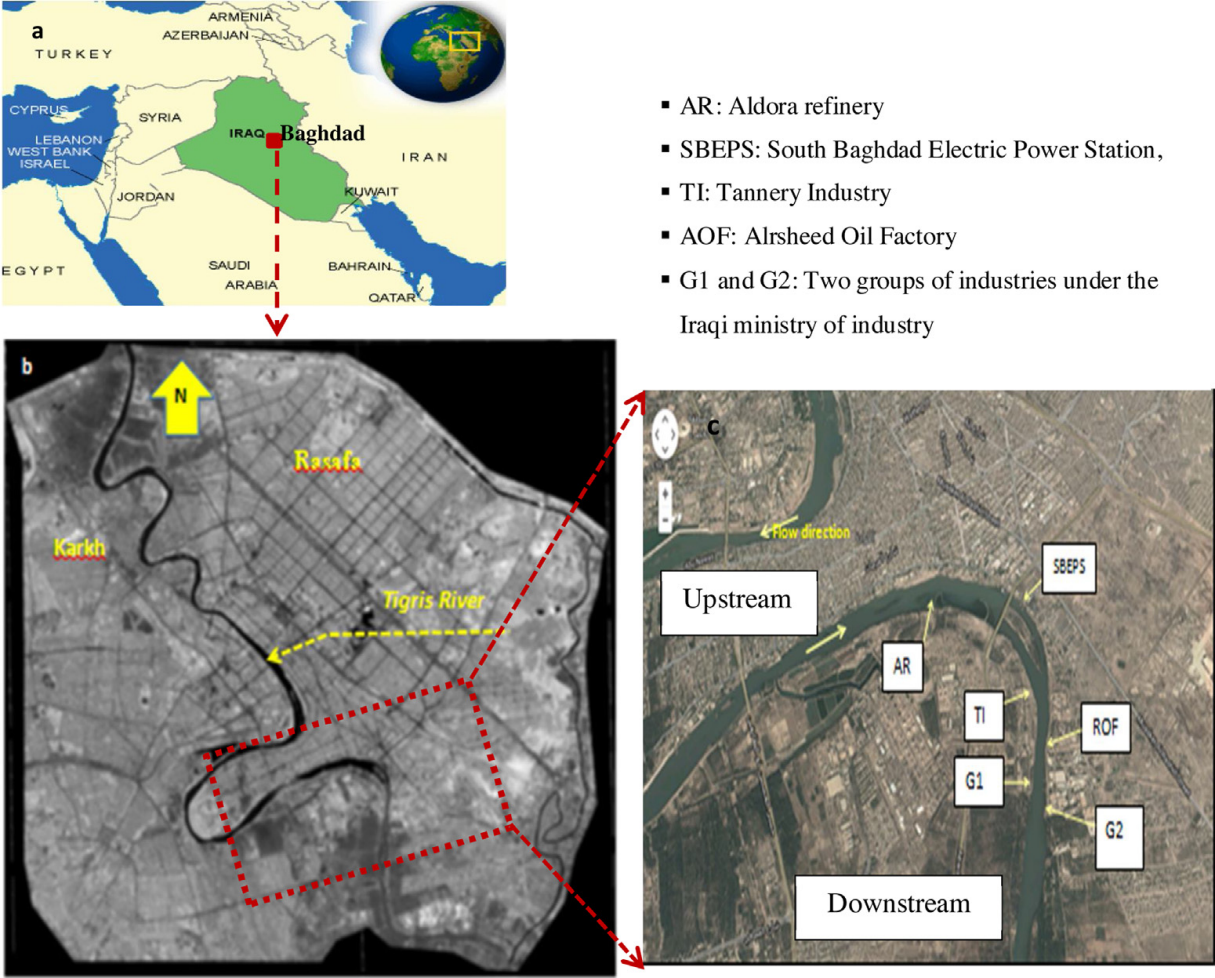
Geographic map of Iraq (a); satellite image for Baghdad (b) [8], and (c) Geographic Map showing study region.

Adapted from Google Maps.

Approximately, the length of Tigris River is 1900 km, the major (77%) part of which flows in Iraq, followed by Turkey (22%) and Syria (1%). The Tigris River is the main river of Baghdad (capital of Iraq), and, by its flow, it divides the city into two parts: the Karkh and Rasafa districts. It is considered as the major source of water for the Baghdad city and its downstream cities. The study region is significant due to the presence of various wastewater drains joining the river there [8]. The majority of its municipal and industrial wastes are discharged directly into the river without adequate treatment, which has polluted the river extensively with organic wastes. Unfortunately, the latest reports suggest that the condition of this river has deteriorated in several regions, leading to worsening of the water quality of the river [8], [9]. Therefore, effective management of this segment of the river is of prime importance. Fig. 1a and b shows a geographic map of Iraq and a satellite image of Landsat7-ETM (2009) corrected by ERDAS 9.2 for Baghdad city, respectively [1], in addition, Fig. 1c is a Google Map showing study region with the locations of six wastewater discharge outfalls.

The quality of the treated wastewater produced from each wastewater treatment plant (WWTP) of the mentioned industries is regularly tested by the Iraq Ministry of Environment. The six wastewater treatment plants (Fig. 1c) are located approximately 2–4 km apart. As mentioned previously, the amount of organic pollution in the discharged wastewater from these six sources was determined in terms of their BOD_5_.

### Modeling and method description

**Fig. 2:**
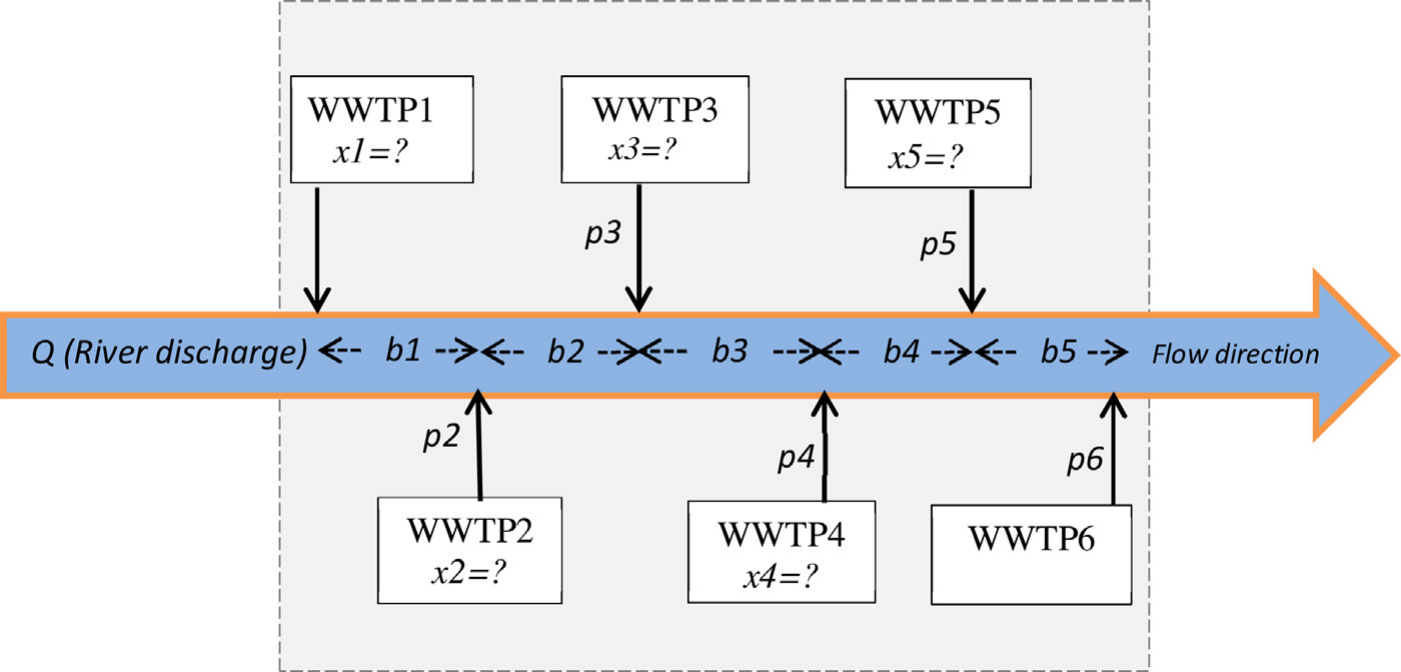
Schematic representation of the input/output parameters of the study problem.

The water quality optimization model adopted herein has as an objective function for determining the best treating efficiency of multi-WWTPs. BOD_5_ concentration of river water was chosen as a guide and an indication test of the water quality. Therefore, in this study, a considerable attention was paid to the determination of the BOD_5_ removal efficiency for each WWTP as well as the maximum allowable BOD_5_ loading in the Tigris River. The latter point depends on the Iraqi standards of rivers water. The most widely used model to solve such problem is LP [13]. For this purpose, the river region was divided into five reaches; each reach was connected to two nearest WWTP outfalls (Fig. 2). To develop and demonstrate the calculations involved in this system, which consisted of six different WWTPs (*i* = 1 to 6), the following definitions and assumptions were considered:

*Qi* = River water flow (m^3^/day) on reach *i* − (*i* + 1), this parameter is stable for all river reaches and varies seasonally; hence in the present study the mean monthly values are considered.

*pi* = BOD_5_ discharge rate from WWTP*i* (g/day). It is worth to mention that water pollution load is the essential input data for establishing water quality management model, and it is an important factor that affects the optimization of wastewater outfall locations [4], [17].

*xi* = optimized efficiency corresponding to each WWTP, which is the determination of its value is considered as an objective function.

*bi* = maximum allowable BOD loading in each reach (g/m^3^). This parameter is defined by the Iraqi Ministry of Environment Standards and found to be ≤30 g/m^3^ (or mg/L). Therefore, this value was fixed for all studied river reaches.

The first question highlighted from the above problem of six WWTPs discharging their wastewater into the same river is “what is the optimization objective function?”. The objective function seeks to minimize the operation efficiency of each plant in which the BOD_5_ loading in the river does not exceed the allowable limit. Thus, the objective function for each month is:

(1) $$minimize\;f=\overset{5}{\underset{i=1}{\sum }}x_{i}p_{i}$$

To solve the objective function that satisfies the BOD_5_ loading requirement in reach 1–2 (river reach between the outfall location of WWTP1 and WWTP2), we must subject the objective function to the following constraints:

(2) p1(1−x1)≤b1Q1

Similarity, the BOD_5_ loading constraint for reach 2–3 can be represented in the following form:

(3) (1−r12)(BOD5dischargerateinreach1−2)+(BOD5dischargerateinreach2−3)≤b2Q2

or

(4) (1−r12)p1(1−x1)+p2(1−x2)≤b2Q2

The coefficient *r12* (<1) represents the fraction of waste removed in reach 1–2 by decomposition which is chosen to be 4%. As the distances between the two WWTP outfalls were approximately equal, the fraction of BOD_5_ removed by decomposition was equal for all five river reaches.

In a similar manner, for reaches 3–4, 4–5, and 5–6, the constriant will be as shown in Eqs. 3.5, 3.6, and 3.7, respectively:

(5) (1−r23)[(1−r12)p1(1−x1)+p2(1−x2)]+p3(1−x3)≤b3Q3

(6) (1−r34){(1−r23)[(1−r12)p1(1−x1)+p2(1−x2)]+p3(1−x3)}+p4(1−x4)≤b4Q4

(7) (1−r45){(1−r34){(1−r23)[(1−r12)p1(1−x1)+p2(1−x2)]+p3(1−x3)+p4(1−x4)}}+p5(1−x5)≤b5Q5

Moreover, the above nonlinear equations system must consist the minimum and maximum allowable treatment efficiencies that can be presented by the following constraint:

(8) lb≤x1,x2,x3,x4,x5≤ub

Where *lb* and *ub* is the minimum and maximum operating efficiencies for each plant. According to the personal contacts with the corresponding engineers of these plants, the minimum and maximum possible plant efficiency was maintained at 50% and 90% under the best conditions, respectively.

The above model equations consider the BOD minimization values in rivers as the primary objective in a multi-objective optimization problem. The intent of a model of this system is to elucidate treatment strategies that can be utilized to decide ways to enhance the quality of a water body in an optimum results. It is noteworthy that, for this case, the proposed model did not attempt to solve problems regarding capacity expansion, wherein a long-term planning horizon was advocated [2]. The problem model equations (Eqs. (1)–(9)) represent a linear equations algorithm that can be solved by using the LP. The function for the solution of the LP system is built in the MATLAB program version (7.9). This function was named “linprog”, which solves a system of linear equation problem.

### Method validation

Table 1 listed the average montly river flow rate (*Q*, m^3^/day); BOD_5_ discharge rate (*p*, g/day), and maximum allowable BOD_5_ loading (*b*, g/m^3^) for all studied reaches. The WWTP1, WWTP2, WWTP3, WWTP4, and WWTP5 were for AR, SBEPS, TI, AOF, and G1 plants, respectively. These data are extremely important to find the appropriate management system according to the above mentioned equations. These data were arranged for each month of the year 2016. It is worth to note that the BOD_5_ concentration upstream of the AR (1st WWTP in the system) was negligible since there were no activities from the river entering point to the Baghdad to the AR outfall. Therefore, the BOD_5_ concentration of the river upstream the system (Fig. 2) was considered to be zero in the modeling program. In addition, and for the sake of completeness, the determined optimized efficiencies were compared with the present operating efficiency for each plant. The present operating efficiency of BOD removal for each plant can be easily calculated by using the following equation:

(9) $$Efficiency\;(\%)=\left(\frac{{\text{BOD}}_{\text{in}}-{\text{BOD}}_{\text{out}}}{{\text{BOD}}_{in}}\right)\times 100$$

**Table 1:** Average monthly river flow rate (m^3^/day); BOD_5_ discharge rate (g/day), and maximum allowable BOD_5_ loading (g/m^3^) for all studied WWTP in 2016.

	Reach 1–2 (WWTP1-WWTP2)			Reach 2–3 (WWTP2-WWTP3)			Reach 3–4 (WWTP3-WWTP4)			Reach 4–5 (WWTP4-WWTP5)			Reach 5–6 (WWTP5-WWTP6)		
Month	*Q1* (m^3^/day)	*p1* (g/day)	*b1* (g/m^3^)	*Q2* (m^3^/day)	*p2* (g/day)	*b2* (g/m^3^)	*Q3* (m^3^/day)	*p3* (g/day)	*b3* (g/m^3^)	*Q4* (m^3^/day)	*p4* (g/day)	*b4* (g/m^3^)	*Q5* (m^3^/day)	*p5* (g/day)	*b5* (g/m^3^)
Jan.	867.92	19600	30	867.92	9720	30	867.92	2310	30	867.92	13760	30	867.92	196	30
Feb.	1171.50	14700		1171.50	13770		1171.50	2380		1171.50	9245		1171.50	232	
Mar.	1607.22	16100		1607.22	17010		1607.22	1330		1607.22	8084		1607.22	240	
April	2054.05	13650		2054.05	11664		2054.05	1708		2054.05	7697		2054.05	308	
May	2059.79	15400		2059.79	14580		2059.79	1617		2059.79	6407		2059.79	220	
Jun.	1324.46	18900		1324.46	10125		1324.46	1470		1324.46	7310		1324.46	240	
Jul.	723.89	19950		723.89	13770		723.89	980		723.89	7869		723.89	244	
Aug.	493.41	20300		493.41	10935		493.41	1071		493.41	8815		493.41	312	
Sep.	412.27	15050		412.27	11259		412.27	1470		412.27	8643		412.27	352	
Oct.	411.35	15400		411.35	9882		411.35	980		411.35	5547		411.35	280	
Nov.	525.56	16100		525.56	13689		525.56	1540		525.56	8084		525.56	252	
Dec.	680.60	17150		680.60	13041		680.60	1960		680.60	7525		680.60	196	

### Comparison of the optimized and present efficiencies

**Fig. 3:**
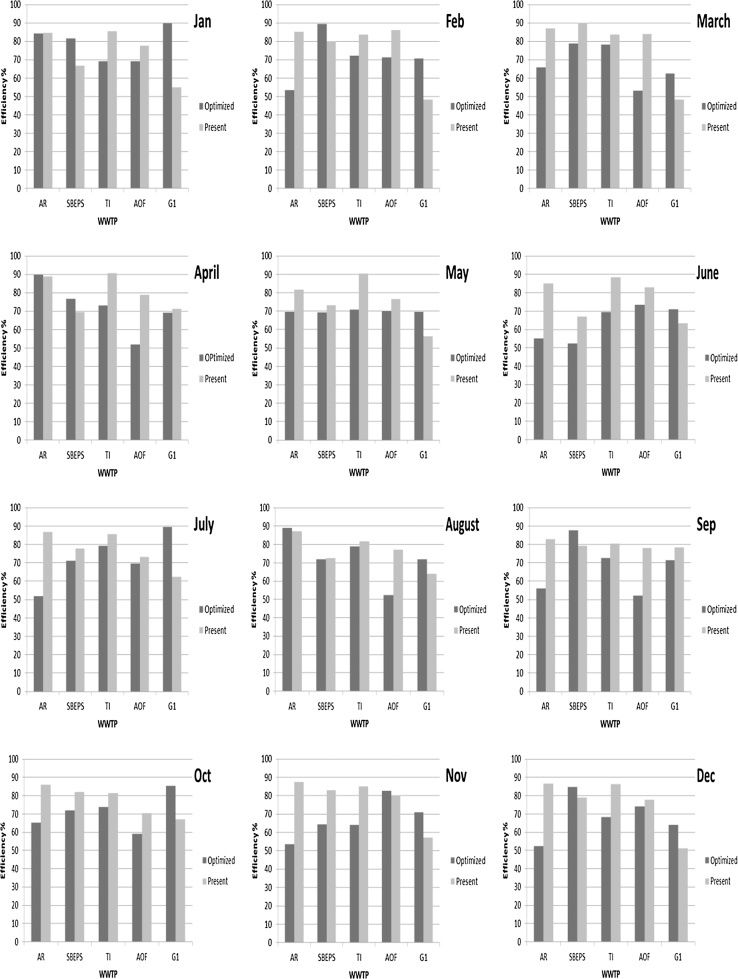
Optimized and present efficiency of five WWTPs for each month in 2016.

The results of variation in the present and optimized operating efficiencies of the studied WWTPs for each month are depicted in Fig. 3. More sophisticated trends to investigate the interplay between these two efficiencies were available in this study. For many cases, the present operating efficiencies for five WWTPs were greater than the optimized values. For the results obtained in January, it can be seen that the reach 2–3 was the only affected river part as the present SBEPS’s operating efficiency was lower than the resultant optimum value owing to high BOD_5_ loading rate in this region. Similarity, great differences were noted between the optimized (90%) and the present (55%) efficiencies of the G1 plant, which may have led to increase in the BOD_5_ concentrations exceeding the allowable limits which is constrained in the mathematical model with <30 mg/L, according to the Iraqi standards. For AR, TI, and AOF WWTPs, the optimized efficiencies were approximately close to or lower than the present working efficiencies. This is a preferred case, especially to the environment, but the surplus treatment costs should be estimated. The maximum optimized efficiency was found for the G1 WWTP because of high accumulative BOD_5_ loading rate in the WWTPs upstream of the G1 WWTP. Minimum optimized operating efficiency was determined for both TI and AOF WWTPs due to high efficiencies were employed upstream them, which resulted mainly for AR and SBEPS WWTPs. Except that the G1 and SBEPS WWTPs is recommended for increasing their removal efficiency to the maximum possible value, the present operating efficiencies of three remaining WWTPs can be accepted in January.

In February, the same results were revealed for January. All the plants are working well, except for the SBEPS and G1, which have a significant difference between the optimized and present efficiencies. This can be reasoned by the BOD_5_ loading rate of the WWTPs upstream G1. In addition, the present efficiency of AR, TI, and AOF WWTPs were higher than those obtained from the optimization results. This result can help us minimize the total treatment cost of the wastewater in this system by applying the management model results. If the operating efficiencies remained within the present condition, the river reaches downstream of the G1 plant will be affected with high organic pollution load. To obtain accurate findings, water quality samples were recommended to be analyzed from several locations downstream of the G1 WWTP outfall.

In March case, it can be seen that all the determined optimized efficiencies were lower than the present values, except for the G1 plant, in which the working efficiency is should be slightly increased from 49% to 61%. It was outlined that in this month, the Tigris River’s flow discharge increases in comparison with that in January and February (Table 1) owing to good water quality and great ability of self-purification process for the organic pollution [15]. For this case of high-removal efficiencies of AR, SBEPS, TI, and AOF WWTPs, it can be concluded that the BOD_5_ concentrations of all reaches will be less than the maximum allowable limit despite that the G1 efficiency is less than the required optimized efficiency.

Irregular fluctuations were noted in the efficiency results for April, wherein all optimized efficiencies were found to be greater than or near to the working present values for the AR, SBEPS, and G1 plants. In the figure corresponding to this month, the AOF can be seen to have minimum optimized efficiency in comparison with the other WWTPs, which can be attributed to the high efficiency suggested for AR, SBEPS, and TI located upstream of the AOF WWTP outfall location. High present and optimized efficiencies were determined for AR and TI WWTPs, respectively. It should be noted that the river flow rate was registered to be high in April (2054 m^3^/s) due to the melting of ice in the northern Iraq [16]. This factor may enhance the self-treatment process for the wastewater discharged into the river [15]. The results shown in Fig. 3 for April revealed that TI and AOF WWTPs working efficiencies can be decreased to 73% and 51%, respectively.

The optimized efficiency values were around 70% in May. In addition, TI WWTP registered the highest present and optimized efficiency values of 90% and 71%, respectively. This difference, in fact, highlighted to the unwanted treatment level was done. From other side, all the WWTPs were found to be working in a safe case, except for the G1 WWTP, in which this plant showed an increased is required in its working efficiency from 56% to 69%. The registered mean flow rate of Tigris River was high (2059.79 m^3^/s). This is a positive sign for diluting the pollution factors like BOD_5_ in the river reaches. According to the results obtained for May, there was no affected reach in the present condition and the same trend of present working efficiencies in April was presented. Conversely, the optimized values for May differed from those of April, which indicates that there are parameters other than river discharge can affect the results of the optimization model.

In June, all the WWTPs were presently working in efficiency above the optimized values, except for the G1 plant which was working close to the optimized efficiencies. Although, it can be say that the system were working in a good case especially when one compared the present and optimized efficiencies for AR WWTP. Despite the slight deviation between the registered river flow rate in May and June, the trend for present efficiencies was found to be similar in these two months. It is clear that if the system was found working at the present efficiencies, the studied system of the river will not be affected due to the high removal efficiencies occurring in the first four WWTPs. Therefore, this findings will lead to a reduction in the BOD_5_ concentrations to values <30 mg/L.

A positive case in July was noted between the two efficiencies for the first four WWTPs. All plants worked at efficiencies greater than required. The difference between the two efficiencies was in a descending manner. For the G1 WWTP, an opposite result was found and the working efficiency for this plant needs to be increased from 62% to 90%. In the present condition and according to the G1 WWTP efficiency, the case of downstream contamination with organic pollution may occur and should be solved to protect the downstream environment by enhancing the G1 plant efficiency. In addition, the optimization program has created a large difference between the optimized and present operation efficiency for AR in July. Actually, AR WWTP was installed for big refinery and discharges a large quantity of wastewater (*p1* values); as a result, the degree of treatment can be significantly decreased. In addition, it is worth mentioning that July is a hot month in Iraq, which may increase the decomposition process of organic material due to the microorganism activity increases with the increasing temperature of the environment [10].

In August, as per the results of the optimization model which highlighted that the AR and G1 WWTPs worked slightly below the optimized efficiency. For these two plants, it was recommended to increase their treating efficiency to agree with the allowable limits of BOD_5_ in the rivers. For AOF, the resulting efficiency was near the 50% as well as SBEPS and TI plants worked at efficiencies near the optimized value. The G1 WWTP should boost its efficiency by 9%, which can be easily performed because the BOD_5_ loading rate of this plant is low as listed in Table 1.

The results obtained in September showed that all the present efficiencies were greater than the optimized efficiency. It is clear that all plants are operating in a high efficiency (nearly 80%). This is a good case for the water quality condition, but not preferable for economic reasons. The optimized efficiency was the highest for SBEPS, while it was lowest for AOF. September, which is also a hot month in Iraq, recorded a flow rate of Tigris River as low as 412 m^3^/s, which may have affected the dissolved oxygen content and as a result the self-purification treatment, especially in the case study region.

The results of October illustrates that the first three WWTPs worked with nearly 82% efficiency while the last two worked with nearly 70% efficiency in October. Furthermore, the results of the programming process demonstrated that the required optimized efficiency was not achieved for the G1 plant. This may cause a significant increase in the BOD_5_ concentrations above the allowable limit, according to the Iraqi standards. For AR WWTP, the present efficiency was greater than the optimized value. The optimized efficiency was lowest for AOF (59%). It could be seen that the G1 was the only plant that needed to work on increasing its efficiency. This could be because the G1 plant is located downstream to four plants and is hence naturally affected by the pollution coming from the upstream plants.

In November, the present efficiencies were found to be greater than the optimized values for AR, SBEPS, and TI WWTPs. For AOF, the two efficiencies were close, but there was a deviation by 14% for the G1 efficiencies. The first three plants operated in efficiencies lower than the present values. The maximum efficiency of treatment was suggested for AR and T1 WWTPs, which was 86%. For this system and to be safe environmentally, it is only recommended for the G1 plant to increase the working efficiency to 71%.

### Effects of the operating parameters

**Fig. 4:**
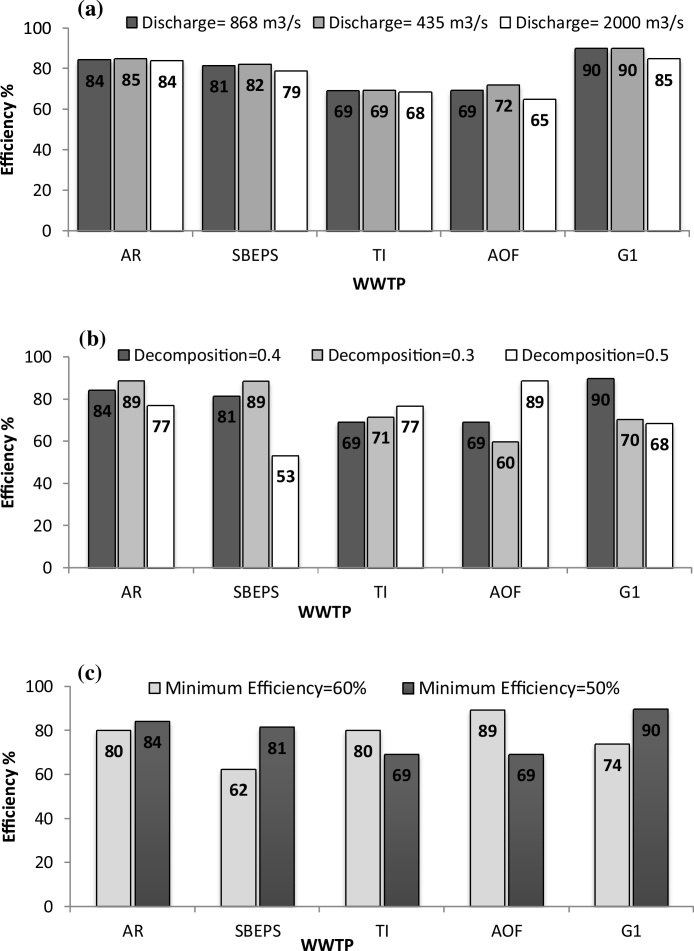
Effects of variation of (a) river discharge; (b) decomposition value, and (c) initial efficiency value on the optimized efficiency of five WWTPs in January.

Further analyses were performed to test the effects of the variation of some of the input parameters. For this purpose, effects of some environmental conditions like river discharge, the fraction of waste removed due to decomposition, and minimum acceptable plant efficiency, were evaluated in this study and the results are illustrated in Fig. 4. It should be noted that the data selected for this study are for January.

Fig. 4a depicts the effects of variation of the Tigris River discharged on the optimized efficiencies of five WWTPs. It can be seen that the required optimized efficiencies at the actual river discharge of 868 m^3^/s were not altered greatly on decreasing the discharge to 435 m^3^/s for all plants. For example for the G1 WWTP, the removal efficiency was almost not affected and remained at 90%. This phenomenon suggests that decrease in the river discharge by half will not alter the dilution property of river toward organic pollution. Therefore, the operated WWTPs should not decrease their operation efficiencies in case of decreasing the river flow in other months to avoid an increase in BOD_5_ concentration in the river above the standardized values. Similarly, for the case of increasing the discharge, it was noticed that, for all WWTPs, the optimizing efficiencies were slightly decreased, especially for the last two WWTPs. This is because increasing the flow rate of the river enhanced the dilution process owing to the improvement in the decomposition of organic matters in the aquatic environment.

The microorganism’s decomposition of organic materials found in wastewater plays an important role in reducing the BOD_5_ concentration of a river. In the present study, the variation of decomposition ability of the Tigris River in January was noted at 0.3 and 0.5 (Fig. 4b). At 0.3 decomposition value, the first three WWTPs should increase their operating efficiencies in comparison with the optimized efficiency calculated at the decomposition rate of 0.4. This phenomenon was cause a decrease in the efficiencies of the last two WWTPs (AOF and G1) due to the improvement of water quality resulting from the treatment efficiencies of upstream plants. For the AR and SBEPS WWTPs, the BOD_5_ loading rate entering this plant was found to be high as compared with other plants. Therefore, these two plants should increase their efficiencies to avoid the increase in the BOD_5_ value in the river to >30 mg/L considering a decrease in the decomposition process from 0.4 to 0.3. For G1, the results was similar to that of AOF plant because it resulted an increase in the first three plants showed improved river condition until the AOF plant. In other words, due to the increase in the operating efficiency of the first three plants (as determined at 0.3 decomposition fraction), the water quality of the system is also improved without increasing the efficiencies of downstream plants. This case can be continued until reaching the downstream G1 outfall.

The increase in the initial operating efficiency value was greatly affected by the operating efficiency, but there was no uniform trend (Fig. 4c). This is because, increasing the minimum removal efficiency from 50% to 60% increase the efficiency of the first two plants. Hence, for the subsequent two plants (TI and AOF), the operating efficiency decreased significantly. Same results as for the first two plants were noted for G1 WWTP.

### Summary

• The use of optimization methods against surface water quality problems can serve as a useful tool to simulate and predict the concentration of pollutant in rivers.
• The data collected of BOD, river flow rate, and plants wastewater discharges were found to significantly vary every month. Therefore, the model results were produced monthly.
• A complex phenomenon to investigate the interplay between optimized and present efficiencies of the studied WWTPs was available in this study.
• In many cases, the optimized determined efficiencies were in values lower than the present efficiencies. This is a positive point as it indicates that the discharge wastewater had BOD_5_ concentration lower than what can be accepted by the river. But, this point is not desired due to the unwanted costs involved.
• It should be noted that the variation in the river flow rates is not an important factor affecting the determination of BOD concentrations in the river and hence its effect on the determined values of operating efficiencies. In many situations, the value of decomposition rate was found to strongly affect the operating efficiencies.
• The variation in the operating efficiency value of the first upstream plant greatly affected the operating efficiency of the downstream plants. In addition, it was found that, G1 WWTP was the only plant that needed to increase its efficiency. This is because G1 was located downstream of the four plants and hence was naturally affected by the pollution arising from the upstream plants.
• There was no constant rank for the investigation on the effects of the natural decomposition on the operating efficiency for each plant.

## Additional information

The warning of environmental issues among the world communities has reached an unforeseeable level. This active awareness is driving our industry to achieve levels of waste treatment performance far beyond those envisioned even as recently as in the last decade [6], [12]. One of the most important and well-known surface water quality parameters is the biochemical oxygen demand (BOD), often measured as 5-day BOD (BOD_5_). Moreover, the ecologists around the world emphasize the importance of BOD as an indicator to assess the degree of organic contamination of receiving water body such as rivers and the efficiency of treatment units [5], [7]. The main sources of this type of pollution in rivers are human and animal liquid wastes that restrict water utilization due to impaired ecosystem health and the necessary treatment expense [14]. Therefore, considering an appropriate management system of wastewater disposal from various activities into the receiving water body is an important process to protect this aquatic environment [11]. Linear programming (LP) is one of the most widely used optimization techniques and perhaps the most effective one. The term LP was coined by George Dantzig in 1947 to describe mathematically the problems in which both the objective function and the constraints are linear [3].

## References

[1] Al-Shami A., Al-Ani N., Al-Shalchi K.T. (2006). Evaluation of environmental impact of Tigris river pollution (between Jadirriya and dora bridges). J. Eng..

[2] Burn D.H., McBean E.A. (1987). Application of nonlinear optimization to water quality. Appl. Math. Modell..

[3] Dantzig G.B., Orden A., Wolfe P. (1998). Generalized simplex method for minimizing a linear form under linear inequality restraints. Pac. J. Math..

[4] Eisele M., Kiese R., Krämer A. (2001). Application of a catchment water quality model for assessment and prediction nitrogen budgets. Phys. Chem. Earth (B).

[5] Fan C., Kao C., Liu Y. (2017). Quantitative characterization of organic diffusion using an analytical diffusion-reaction model and its application to assessing BOD removal when treating municipal wastewater in a plug flow reactor. Water Res..

[6] Fu F., Wang Q. (2011). Removal of heavy metal ions from wastewaters: a review. J. Environ. Manage..

[7] Hammer J., Hammer (2012). Water and Wastewater Technology.

[8] Ismail A.H., Abed G.A. (2013). BOD and DO modeling for Tigris River at Baghdad city portion using QUAL2K model. J. Kerbala Univ..

[9] Issa I.E., Al-Ansari N.A., Sherwany G., Knutsson S. (2014). Expected future of water resources within Tigris-Euphrates rivers basin, Iraq. J. Water Resour. Prot..

[10] Li L., Zheng B., Liu L. (2010). Biomonitoring and bioindicators used for river ecosystems: definitions, approaches and trends. Procedia Environ. Sci..

[11] Saremi A., Sedghi H., Manshouri M., Kave F. (2010). Development of multi-objective optimal waste model for Haraz river. World Appl. Sci. J..

[12] Sulaymon A., Mohammed A., Al-Musawi T. (2013). Removal of lead, cadmium, copper, and arsenic ions using biosorption: equilibrium and kinetic studies. Desalin. Water Treat..

[13] Taha H.A. (2007). Operations Research: an Introduction.

[14] Tchobanoglous G., Burton F.L., Stensel H.D. (2003). Wastewater Engineering: Treatment and Reuse.

[15] Tian S., Wang Z., Shang H. (2011). Study on the self-purification of Juma river. Procedia Environ. Sci..

[16] USGS (U.S. Geological Survey) (2000). Quality-Assurance Plan for Water-Quality Activities in the North Florida Program Office, Florida District. Open-File Report 00-426. http://fl.water.usgs.gov.

[17] Xing K., Guo H., Sun Y. (2004). Simulation of non-point source pollution in Lake Dianchi basin based on HSPF model. China Environ. Sci..

